# Electrospun PVDF/aromatic HBP of 4th gen based flexible and self-powered TENG for wearable energy harvesting and health monitoring

**DOI:** 10.1038/s41598-023-50231-z

**Published:** 2023-12-19

**Authors:** Ramadasu Gunasekhar, Mohammad Shamim Reza, Kap Jin Kim, Arun Anand Prabu, Hongdoo Kim

**Affiliations:** 1 Department of Chemistry, School of Advanced Sciences, Vellore Institute of Technology, Vellore, 632014 India; 2 Department of Advanced Materials Engineering for Information and Electronics, College of Engineering, Kyung Hee University, Yongin-si, Gyeonggi-do 17104 Republic of Korea

**Keywords:** Chemistry, Materials science

## Abstract

In recent times, high-performance wearable electronic devices that can transform mechanical force into electrical energy for biomedical monitoring applications are receiving an increasing amount of attention. In the present study, we focused on a flexible, self-powered and wearable triboelectric nanogenerator (TENG) based on electrospun polyvinylidene fluoride (PVDF)/aromatic hyperbranched polyester of 4th generation (Ar.HBP-G4, 0–40 wt.-% w.r.t. PVDF content) blend nanoweb as tribo-negative layer and melt-blown thermoplastic polyurethane (TPU) as tribo-positive layer for energy harvesting and human health monitoring applications. Among the varying Ar.HBP-G4 content used, incorporation of Ar.HBP-G4 (10 wt.-%) in PVDF (P-Ar.HBP-G4-10) showed higher increase in the triboelectric output voltage when compared to pristine PVDF and other Ar.HBP-G4 weight ratios. The optimized P-Ar.HBP-G4-10/TPU based TENG exhibited a peak-to-peak voltage (V_*p-p*_) of 124.4 V under an applied load of 9.8 N and frequency 1 Hz which is superior to many other TENGs reported elsewhere. Higher triboelectric performance of P-Ar.HBP-G4 blend based TENG compared to that of neat PVDF is attributed to the effect of Ar.HBP-G4-10 in enhancing the degree of crystallinity and polar *β*-crystalline phase content (98.3%) in PVDF. The ability of the TENG to power up portable electronic devices is demonstrated when it is powered for 750 s while connected through a capacitor and a rectifier, and the TENG was able to operate 45 light-emitting diodes directly. Evaluation of the triboelectric output of the TENG device attached to different parts of the human body reveal significantly better output voltage and sensitivity for human health monitoring. The results of this work pave a new way to develop TENG based on P-Ar.HBP-G4 nanowebs for sustainable energy generation and wearable healthcare monitoring systems.

## Introduction

Currently, the growing need for innovative methods in disease diagnosis, therapy and health assessment has rendered human physiological signal monitoring progressively more significant^1–10^. While operating wirelessly, traditional monitoring systems/sensors are usually powered by rechargeable batteries, which makes the sensors heavy and possesses a limited capacity^11^. There are many different energy harvesters that can be incorporated into the system to scavenge energy from the working environment in order to address the power supply issue, including piezoelectric nanogenerators^12–17^, electromagnetic generators^18,19^, thermoelectric generators^20,21^ and solar cells^22,23^. In recent times, triboelectric nanogenerators (TENGs), an emerging technology have drawn a lot of attention due to its ease of manufacturing, flexible design and high energy conversion efficiency that can harvest mechanical energy from their surroundings. More importantly, TENGs have the ability to operate as self-powered sensors as well as the added advantage of generating high output signals and easy handling. However, it is important to improve the TENGs sensitivity of the signals generated on the human body for physiological monitoring using different materials^24–28^.

Generally, the working principle of TENGs is based on two dielectric materials with different triboelectric polarities coming in contact (or come into friction), and the combined impact of electrostatic induction and triboelectrification takes place^1,5,29–32^. Structure and composition of the triboelectric material’s surface have a significant impact on the output of TENGs, since triboelectrification is a surface-charging effect. By increasing the surface area or the disparity in triboelectric polarity between the layers, surface modification can enhance the surface charge density. Furthermore, another key factor affecting the triboelectric performance is the dielectric constant of a triboelectric material^33–35^.

Though almost all the materials possess triboelectric properties, a newly developed triboelectric material with unique micro- and nanostructure may enhance the TENG output suitable for commercial applications. Evidently, there are different materials including metals (Au and Al)^36,37^, conductive polymers (polypyrene, polyaniline, polyvinylidene fluoride (PVDF))^4,31,38–40^, inorganic semiconductors (TiO_2_, ZnO)^41,42^ and insulating polymers (polytetrafluoroethylene, nylon, polydimethylsiloxane)^43,44^ that have been employed as triboelectric materials in the fabrication of TENGs. Among them, PVDF is a semi-crystalline and electro-active polymer, and exhibit good chemical and mechanical stability, high thermal strength and stability at high electric field^4^. These significant properties have made PVDF a promising material for many different applications, including super-capacitors, actuators, transducers and batteries in the field of electronics engineering.

Electrospinning is the most widely used technique for the construction of fiber-structured nanogenerators. Electrospinning is a simple, low-cost and versatile method to produce ultrathin fibers from a rich variety of materials including polymers, composites, and ceramics^17,32,45–47^. A range of unique properties is shown by electrospun nanofibers (NFs) such as high surface area, one-dimensional morphology, hierarchically porous structures and extraordinary strength.

Our research group is mainly focused on studying the piezo and triboelectric characteristics of electrospun PVDF blended with varying additives^4,17,48–50^. In our earlier study using aromatic hyperbranched polyester of 3rd generation (Ar.HBP-G3) as the blend component (0–40 wt.-%) in PVDF^48^, the PVDF-Ar.HBP-G3-10 (herein mentioned as P-Ar.HBP-G3-10) based TENG device exhibited peak-to-peak voltage (*V*_p−p_) output of 107 V which is almost ten times higher than that achieved for neat PVDF (12 V). In the present study, P-Ar.HBP-G4 (0–40 wt.-%) blend NFs were prepared by electrospinning and analyzed using spectral and microscopic techniques to confirm their structural and morphological characteristics. The optimized P-Ar.HBP-G4-10 based TENG exhibited *V*_p–p_ of 124.4 V which is 16% greater than that achieved for P-Ar.HBP-G3-10 case. Improved triboelectric performance of P-Ar.HBP-G4 blend based TENG is attributed to the effect of higher branching units and more number of terminal OH groups in Ar.HBP-G4-10 structure. There is a need for better understanding of triboelectric effect of the materials used i.e., the correlation of charge density with the chemical structure of materials and interaction between the triboelectric layers. Compared to Ar.HBP-G3, the availability of higher OH terminal groups in Ar.HBP-G4 may have a greater influence on the electron affinity of the TN layer, which in turn could result in improved TENG device performance. The ability of the TENG to power up portable electronic devices is demonstrated by operating 45 light-emitting diodes (LEDs) directly. Evaluation of the triboelectric output of the TENG device attached to different parts of the human body reveal significantly better output voltage and sensitivity for human health monitoring. The results are reported in detail in the following sections.

## Methods

### Materials and preparation

#### Preparation of P-Ar.HBP-G4 electrospun NFs

**Figure 1:**
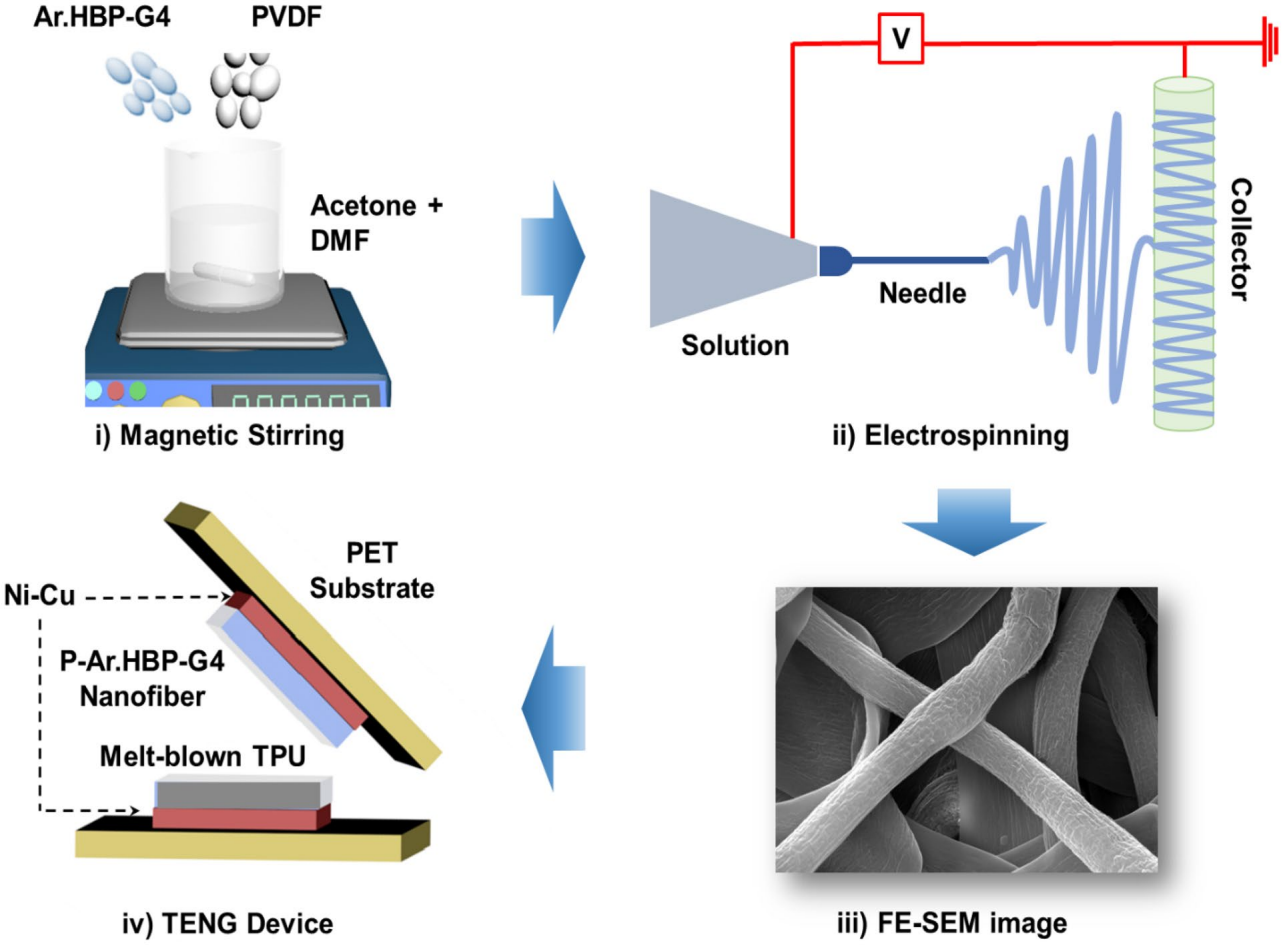
Schematic illustration of the preparation and electrospinning of P-Ar.HBP-G4 solution followed by TENG fabrication: (i) P-Ar.HBP-G4 solution blending by magnetic stirring, (ii) Electrospinning processes, (iii) FE-SEM image of electrospun P-Ar.HBP-G4-10 NF and (iv) Components used in the TENG device.

*N*,*N*-Dimethyl formamide (DMF), acetone and PVDF powder (M_w_ ~ 370,000) were purchased from Sigma-Aldrich, USA. Synthesis and spectral characterization of Ar.HBP-G4 is given in Figs. S1 and S2, respectively in the supplementary material. Initially, 1.6 g of P-Ar.HBP-G4 with varying Ar.HBP-G4 content (0–40 wt.-%) was dissolved in a 10 mL mixture of DMF and acetone (6:4) and magnetic stirred for 5–6 h at 50 ℃. The prepared solution was then loaded in a 10 mL syringe pump mounted with a 23-gauge sharp needle. The parameters followed during electrospinning process are: needle-to-collector distance 15 cm, flow rate 1.2 mL/h, drum-collector speed 60 rpm and applied voltage 20 kV. To ensure the complete removal of residual solvents in P-Ar.HBP-G4 nanofiber mat, the samples were kept under closed vacuum at 60 ℃ for overnight. The nanoweb layer was peeled from the collection mat and used for characterization and further fabrication of the TENG. Figure 1(i and ii) illustrates the preparation of electrospun nanofiber.

#### Fabrication of P-Ar.HBP-G4 based TENG

In the fabrication of TENG, electrospun P-Ar.HBP-G4 NFs (FE-SEM image of Fig. 1(iii)) act as tribo-negative (TN) material and as-received melt-blown thermoplastic polyurethane (TPU) film act as tribo-positive (TP) material, and herein considered as the triboelectric pair. To be used as an active material in the fabrication of TENG, electrospun P-Ar.HBP-G4 and TPU layers of size 20 mm × 20 mm were stacked on one another carefully. A custom-designed adhesive Ni-Cu based conductive fabric serving as top and bottom electrodes were used to sandwich the triboelectric pair on both sides. Further, Cu wires were soldered to the adhesive electrodes. Finally, for additional protection and durability, a commercial PET sheet was used to encapsulate the TENG. Vertical contact-separation mode is used for the fabrication of TENG device and its working mechanism is shown in Fig. 1(iv). Triboelectrification occurs by the contact and separation process of the TN and TP layers and the fabricated TENG is studied for wearable and portable electronic applications.

### Characterization

To confirm the enhancement of *β*-phase in PVDF with the addition of Ar.HBP-G4 (0–40 wt.-%), FTIR-ATR spectroscopy (PerkinElmer Spectrum 400) was used over a range of 400–4000 cm^−1^ with a resolution of 4 cm^−1^. X-ray Diffractometer (XRD, Bruker D8 Advance, Germany) with Cu-Kα radiation source equipped with a 1-dimensional fast detector was used to collect the XRD patterns. Changes in surface morphology and fiber diameter were observed using Field-Emission Scanning Electron Microscopy (FE-SEM, Thermo Fisher FEI QUANTA 250 FEG) at an acceleration voltage of 10 kV. Elemental composition was analyzed using Energy Dispersive X-Ray Spectroscopy (EDS) attached to FE-SEM instrument. Raman spectra **(**BRUKER RFS 27 Multi-RAM FT-Raman Spectrometer**)** were recorded with an excitation wavelength of 532 nm. The triboelectric output performance of the TENGs including the open-circuit voltage (*V*_OC_) and short-circuit current (*I*_SC_) were investigated using a digital phosphor oscilloscope (DP04104, Tektronix) connected to electrometer (Keithley 6514) with an input impedance of 40 MΩ. Using a custom-made dynamic setup, electrical output performance was investigated as a function of load (10 N) and frequency (1 Hz). BIOPAC MP150 data acquisition system (BIOPAC, Inc., USA) with an input resistance of 100 MΩ and a signal gain of 20 dB connected to a measurement specialist Piezo Film Lab Amplifier was used to measure the triboelectric output performance of the TENG for healthcare monitoring studies. The captured data is wirelessly communicated to the portable device (computer/mobile phone) using Acknowledgment software during the measurement.

## Results and discussion

### Working mechanism

**Figure 2:**
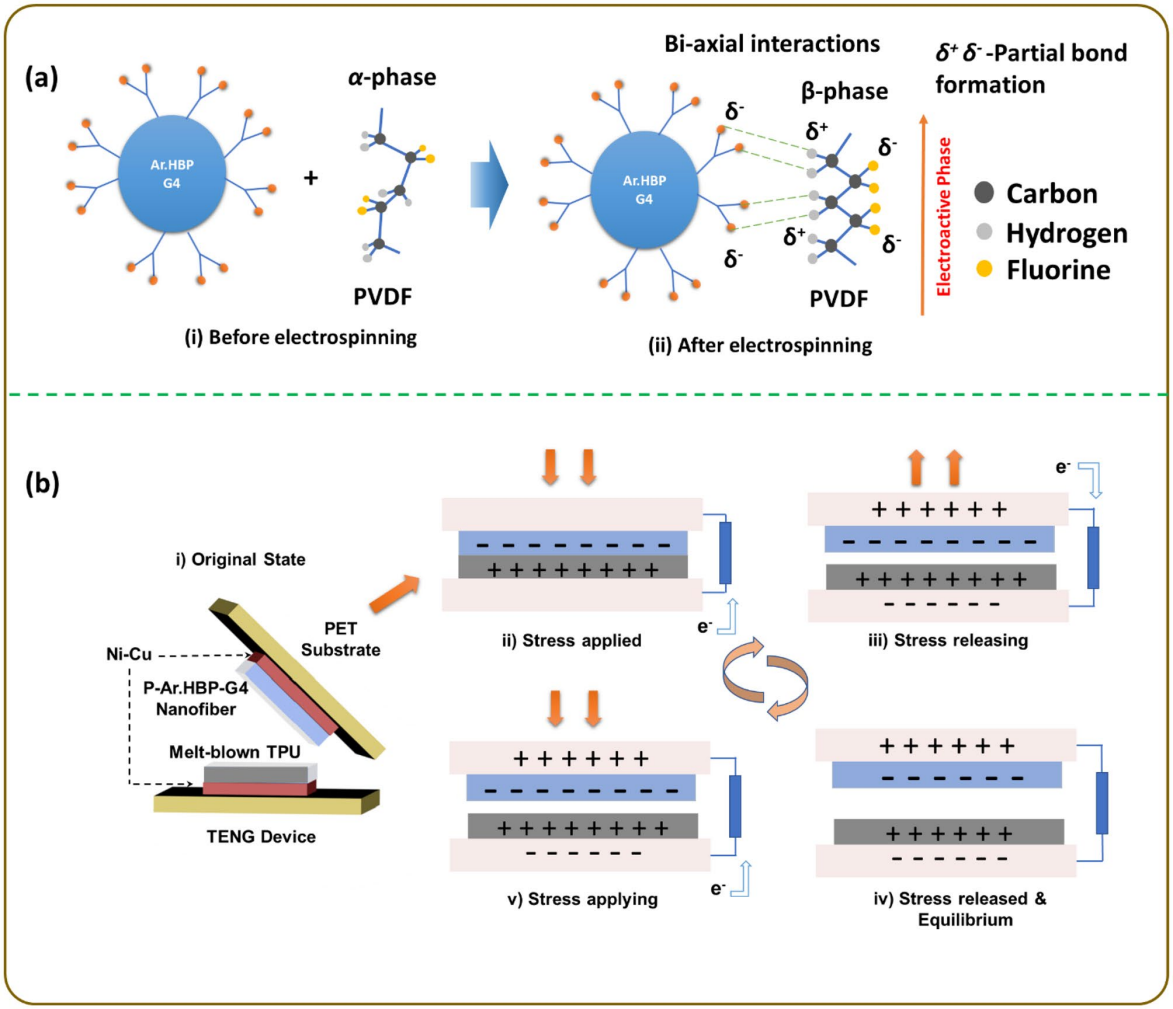
(**a**) Mechanism of *β*-phase formation in P-Ar.HBP-G4 NFs and (**b**) Schematic illustration of TPU and P-Ar.HBP-G4 triboelectric mechanism.

The proposed mechanism for formation of *β*-phase on the P-Ar.HBP-G4 surface is shown in Fig. 2a. Ar.HBP-G4 has a significant role to play towards improving the *β*-phase in PVDF. Similarly, the interfacial interactions of Ar.HBP-G4 can align the C–F dipoles PVDF chains to form crystals on the surface in an all-trans form, thereby enabling the conversion of localized non-polar *α*-phase to polar *β*-phase. Ar.HBP-G4 serves as a nucleating agent in this circumstance, offering basic units for the creation of PVDF’s *β*-phase fragment through potent interaction at the interface^6^. As shown in Fig. 2a, owing to the strong interaction between F atoms of PVDF and H atoms in hydroxyl groups on the Ar.HBP-G4 surface, dipoles of –CH_2_/–CF_2_ in PVDF tend to align their F atoms towards the Ar.HBP-G4 at the P/Ar.HBP-G4 interface.

The device's working mechanism is shown in our earlier work^4,48^. As shown in Fig. 2b(i), in the original state before applying external pressure, the triboelectric materials meet with each other partially. Due to the roughness of P-Ar.HBP-G4 NFs and uneven surface morphology of TPU, there exists a tiny space in between them even though they are partly in contact with each other. As shown in Fig. 2b(ii) under stress applied state, both the tribolayers undergo maximal contact as they are squeezed together when the external force is applied on the TENG. High potential differences are produced as a result of the increase in both negative and positive charges on the surfaces caused by an increase in the area of contact between the frictional layers. Both the layers of triboelectric materials are separated slowly and potential differences are created in between two electrodes when the external force is removed. As shown in Fig. 2b(iii) under stress releasing state, the electrons flow from top electrode to the bottom electrode to maintain the electrical equilibrium. As shown in Fig. 2b(iv) under stress released and equilibrium state (when released fully), this phenomenon will continue until both corresponding tribolayers and the electrode attain opposite and equal charges. As shown in Fig. 2b(v) under stress applying state, to maintain the equilibrium, the tribolayers come closer and electrons will start to flow in reverse from the bottom to the top by the further application of external load. Throughout the measurement, this cyclic procedure is repeated which results in a constant alternate output^5,29,31^.

The output performance of TENGs is influenced by several important factors like selection of materials, surface morphology of the NFs and environmental agents. The triboelectric effect and level of contact electrification performance are based on materials that come into contact and the surface transfer of electrons or ions between these two materials. Each material has its own ability to lose or gain electrons during the contact electrification process. This ability can be found in a list of materials (triboelectric series in Fig. S3) where polarity and amount of charge for each material is an important factor. For example, a material like PVDF having fluorine functional groups can attract as many electrons as possible from their counterparts during electrification. On the other hand, PU is positively charged during electrification. Both materials are placed far enough apart in the triboelectric series, which influence their high triboelectric potential on contact.

### Physical and structural characterization

**Figure 3:**
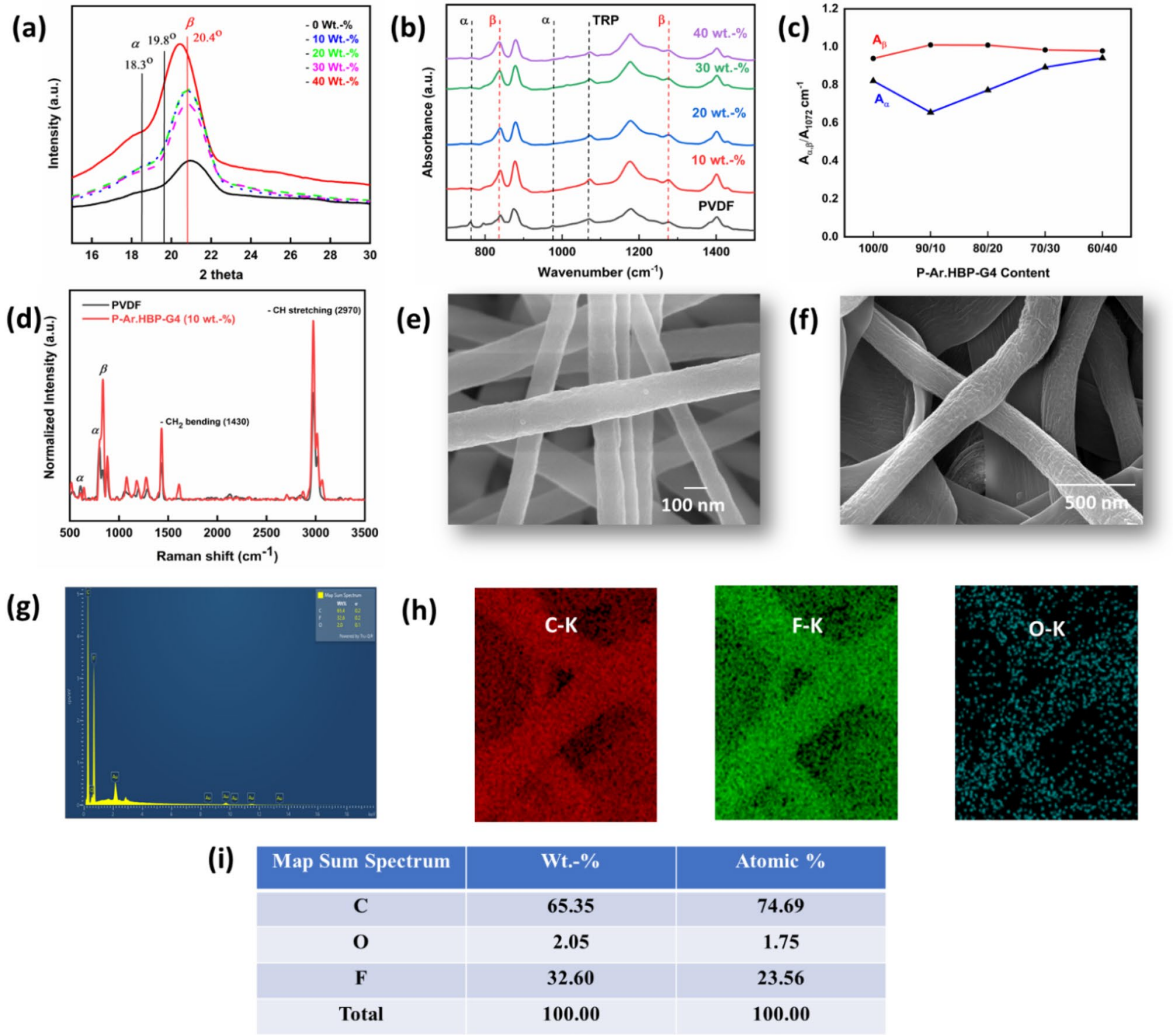
Electrospun P-Ar.HBP-G4 (0–40 wt.-%) blended NFs: (**a**). XRD pattern, (**b**) FTIR spectra, (**c**) Computed *β*-phase content variability data extracted from (**b**), (**d**). Raman spectra, (**e**, **f**) FE-SEM images of neat PVDF and P-Ar.HBP-G4-10 NFs, respectively, (**g**) Map spectrum of elements in P-Ar.HBP-G4-10 NF, (**h**) EDS images of Carbon, Fluorine and Oxygen elements present and (**i**) Their elemental mapping data.

Due to their higher triboelectric sensitivity, PVDF fibers with a significant proportion of *β*-phase are more desirable. Therefore, the effect of Ar.HBP-G4 upon PVDF crystalline forms was investigated using XRD and FTIR measurements. XRD patterns of P-Ar.HBP-G4 (0–40 wt.-%) are shown in Fig. 3a. The electrospun pristine PVDF show two crystalline peaks at 2θ = 18.3° and 19.8°, which are related to the (110) and (020) *α*-phase crystalline lattice planes, respectively. Similarly, P-Ar.HBP-G4 main peak is observed at 2θ = 20.6°, which corresponds to the (102) crystalline plane for electroactive *β*-phase^14,48^.

FTIR results of P-Ar.HBP-G4 (0–40 wt.-%) blend NFs are shown in Fig. 3b. The characteristic FTIR absorption bands of P-Ar.HBP-G4 (0–40 wt.-%) NFs corresponding to *α-*phase is observed at 763 and 976 cm^−1^, *β-*phase at 841 and 1276 cm^−1^ and *γ-*phase at 1234 cm^−1^. FTIR results are summarized in the Table 1. The electroactive *β-*phase crystalline % in NFs is analyzed using the following equation:^49,50^

$$F\left(\beta \right){=A}_{\beta }/{A}_{TRP} \& F\left(\alpha \right){=A}_{\alpha }/{A}_{TRP}$$

where, *A*_*α*_*, A*_*β*_ and* A*_*TRP*_ are the intensities of the absorptions peak at 763, 1276 cm^−1^ and 1072 cm^−1^, respectively. Figure 3c shows the quantitative plot of *α*- (763 cm^−1^) and *β*- (1276 cm^−1^) phases *versus* the TRP. It reveals that the Ar.HBP-G4-10 sample shows higher *β*-crystallinity (98.3%) among the total crystalline content compared to other samples.

**Table 1:** FTIR spectral regions of PVDF based electrospun NFs.

Phase	Wavelength (cm^−1^)
*α* (non-polar)	763 & 976
*β* (polar)	841 & 1276
*γ* (polar)	1234
TRP (thickness relative peak)	1072

During the electrospinning process, *α-* to *β-*crystalline phase transformation takes place due to the nucleation effect of Ar.HBP-4 addition to PVDF. The cross-surface links between PVDF dipoles and Ar.HBP functional groups results in enhanced *β*-phase content and decreasing *a*-phase, further aided by the mobility and architecture of –CH_2_/–CF_2_ dipoles. As a result, when compared to neat PVDF NFs, the P-Ar.HBP-G4 NFs exhibits more *β*-phase content. Similarly, *β*-phase content is lowered (Fig. 3c) with increasing Ar.HBP-G4 content beyond 10 wt.-% due to agglomeration as well as inhibition of the mobility of PVDF chain links. As a result of Ar.HBP-G4 aggregation at the interface, induced charges migrate longitudinally and eventually neutralize the charges in the fiber, which lowers the polarizability of PVDF nanofibers.

In this work, we propose a realistic approach to prepare high *β-*phase content in PVDF matrix using Ar.HBP-G4 as filler. Due to the high coercive force, the addition of Ar.HBP-G4 significantly increased the *β*-phase content in PVDF, which notably impacts the performance output of the TENG. Negative charge on the terminal -OH groups of Ar.HBP-G4 establish a bond with the positive CH_2_ of PVDF via ionic dipole interactions, resulting in preferential development of the polar *β*-crystalline phase in PVDF. From XRD and FTIR data, Ar.HBP-G4-10 doping in PVDF stimulated the formation of the polar *β*-phase, which in turn enhanced the TENG performance. Higher triboelectric performance of P-Ar.HBP-G4 blend based TENG compared to that of neat PVDF is attributed to the effect of Ar.HBP-G4-10 in enhancing the degree of crystallinity and polar *β*-crystalline phase content (98.3%) in PVDF.

Raman spectroscopy results are shown in Fig. 3d. The peaks and shifts of the Raman spectra can be used to confirm the intercalating of polymer chains with Ar.HBP-G4 and the integration of Ar.HBP-G4 into the polymer matrix. The Raman spectrum of the pristine PVDF electrospun NFs as shown in Fig. 3d revealed peaks corresponding to its *α*-phase at 610 and 795 cm^−1^ and *β*-phase at 840 cm^−1^. In contrast, the Raman spectrum of P-Ar.HBP-G4-10 was dominated by signals for –CH_2_ bending at 1430 cm^−1^ and C–H stretching at 2970 cm^−1^, consistent with π–electron delocalization and a degree of structural order in Ar.HBP-G4-10. Shear forces generated during electrospinning of PVDF chains result in the solution jet's rapid stretching due to confinement at the nanoscale^32^. The high alignment of Ar.HBP-G4 along the NF can be attributed to the synergistic effect of the electric field. From Fig. 3d, it is manifested that the diameter of P-Ar.HBP-G4 NFs has increased as the Ar.HBP-G4 is implanted within the PVDF. The significant changes in Raman spectra upon the incorporation of Ar.HBP-G4-10 hinted at a corresponding change in the triboelectric output.

It is essential to confirm the electrospun NFs favorable morphology such as nanofiber formation with uniform fiber diameter, close packing density, and without any bulb distortion along the fiber length prior to fabricating the TENG device. From FE-SEM images of electrospun P-Ar.HBP-G4 (0 and 10 wt.-%) as shown in Fig. 3e,f, the TN fibers are evenly distributed with an average fiber diameter of 200 nm. FE-SEM images show a non-linear, root-shaped fiber formation that made the nanofiber highly elastic. Due to this effect, the triboelectric properties of flexible TENG device can be enhanced. The elemental mapping images are shown in Fig. 3g–i. FE-SEM analysis revealed that adding Ar.HBP-4 can significantly enhance the output performance of the film when compared to pristine PVDF. This is due to the fact that amount of fluorine element on the surface was decreasing continuously which is replaced by hydrogen and oxygen elements from the addition of Ar.HBP-G4 in PVDF^4,48^.

### Triboelectric output

**Figure 4:**
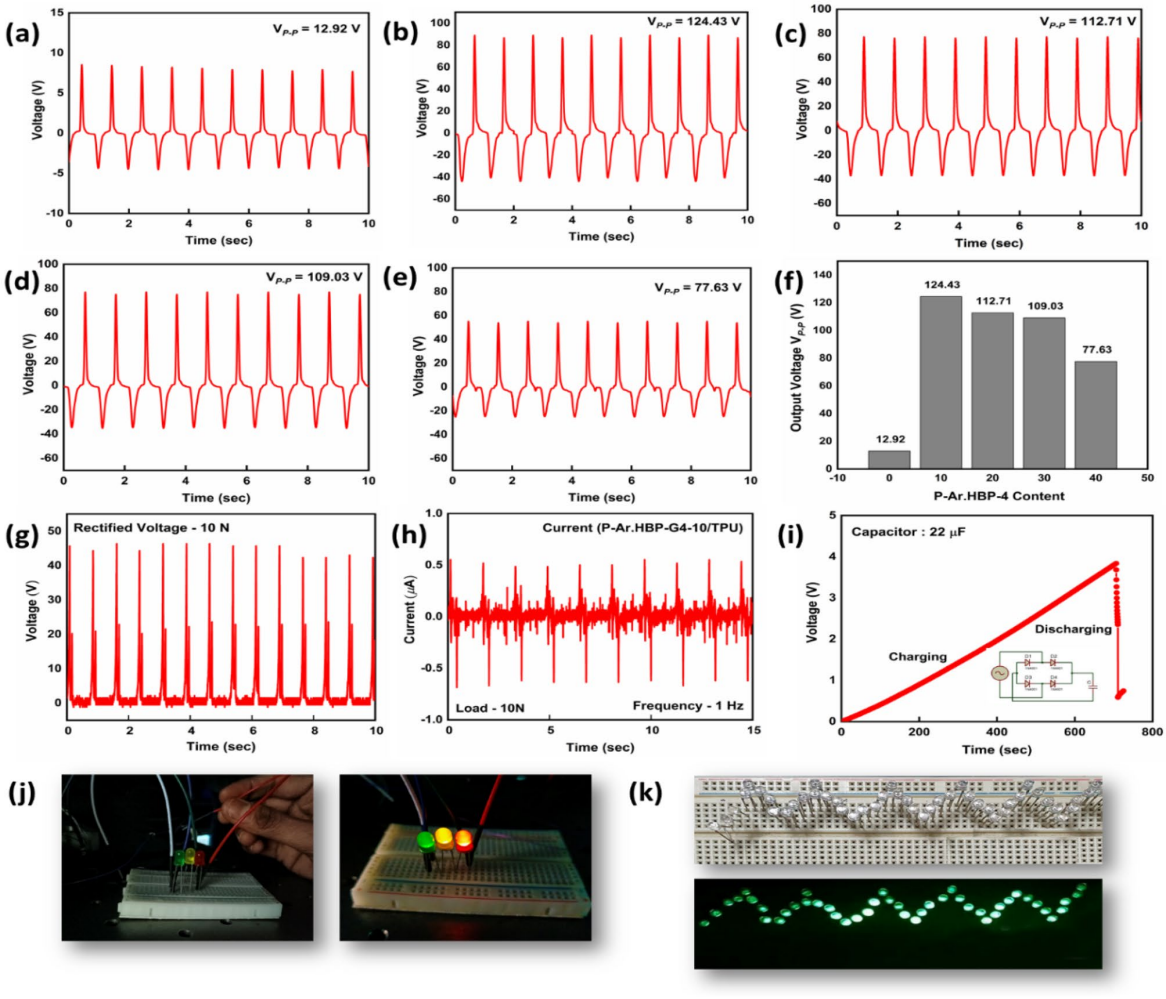
(**a**–**e**) Time dependent V_*OC*_ graphs of P-Ar.HBP-G4/TPU (0–40 wt.-%), (**f**) Quantitative V_*OC*_ of the peak-to-peak (V_*p−p*_) measurements extracted from (**a**–**e**), (**g**) Rectified voltage, (**h**) I_*SC*_ graph of P-Ar.HBP-G4-10/TPU under constant frequency (1 Hz) and load (10 N), (**i**) P-Ar.HBP-G4-10/TPU device energy storage with 22 μF capacitor with charging and discharging cycles (inset circuit diagram for portable device application), (**j**) Images of 3 different colour LEDs and (**k**) Images of 45 LEDs before and after connecting P-Ar.HBP-G4-10/TPU based TENG.

The output performance of electrospun P-Ar.HBP-G4/TPU is shown in Fig. 4a–e with different ratios of Ar.HBP-G4 (0–40 wt.-%) at a constant frequency (1 Hz) and load (9.8 N). The results clearly show that with the enhancement of polar *β-*phase as confirmed earlier from FT-IR and XRD results, the *V*_OC_ values of P-Ar.HBP-G4/TPU increased significantly compared to that of PVDF/TPU. Pristine PVDF/TPU based TENG exhibited 12.9 V, whereas enhanced electrical output performance of 124.4 V, 112.7 V, 109 V and 77.6 V were achieved for 10, 20, 30 and 40 wt.-% Ar.HBP-G4, respectively. Figure 4f shows the quantitative *Vp-p* data extracted from Fig. 4a–e.

From the above results, the output performance of P-Ar.HBP-G4-10/TPU based TENG shows 9.68 times higher output voltage than PVDF/TPU. The better performance of P-Ar.HBP-G4-10 among the varying HBP content is already discussed in the previous section. The increase in the output voltage for P-Ar.HBP-G4-10/TPU TENG compared to PVDF/TPU based TENG may be attributed to the ordering of PVDF and Ar.HBP-G4 in the electrostatic sequence and also the strong ability to generate charge during the friction process. In the case of PVDF/TPU, a significant portion of the charges generated throughout the friction process were quickly neutralized in the air rather than sensed by the electrode material for generating the voltage. Consequently, the excellent electrical performance of P-Ar.HBP-G4-10/TPU can be attributed to higher ability for charge storage coming from Ar.HBP-G4. Therefore, the P-Ar.HBP-G4-10/TPU is optimized for further studies, including wearable and portable electronics.

### Energy harvesting applications

The rectified voltage was stored using a capacitor before connecting any portable electronics. At an operating frequency of 1 Hz and load of 10 N, the P-Ar.HBP-G4-10/TPU rectified voltage is 48 V (Fig. 4g) and the short-circuit current (*I*_SC_) is about 1.1 μA (Fig. 4h). The rectified voltage was retained in a capacitor for approximately 5 s with capacitance value of 10 μF and stored a maximum voltage of 3.7 V. Figure 4i shows the charging and discharging curves, and the circuit diagram is shown in the inset of Fig. 4i. To prove the viability of the P-Ar.HBP-G4-10/TPU based TENG device as a direct power source without any rectifier and energy storage systems, it is successfully used for powering commercial LEDs connected in series. The TENG device efficiently operated 3 LED bulbs of three different colors (RGB) and 45 LED bulbs directly as shown in Fig. 4j,k.

### Long-term stability measurements

**Figure 5:**
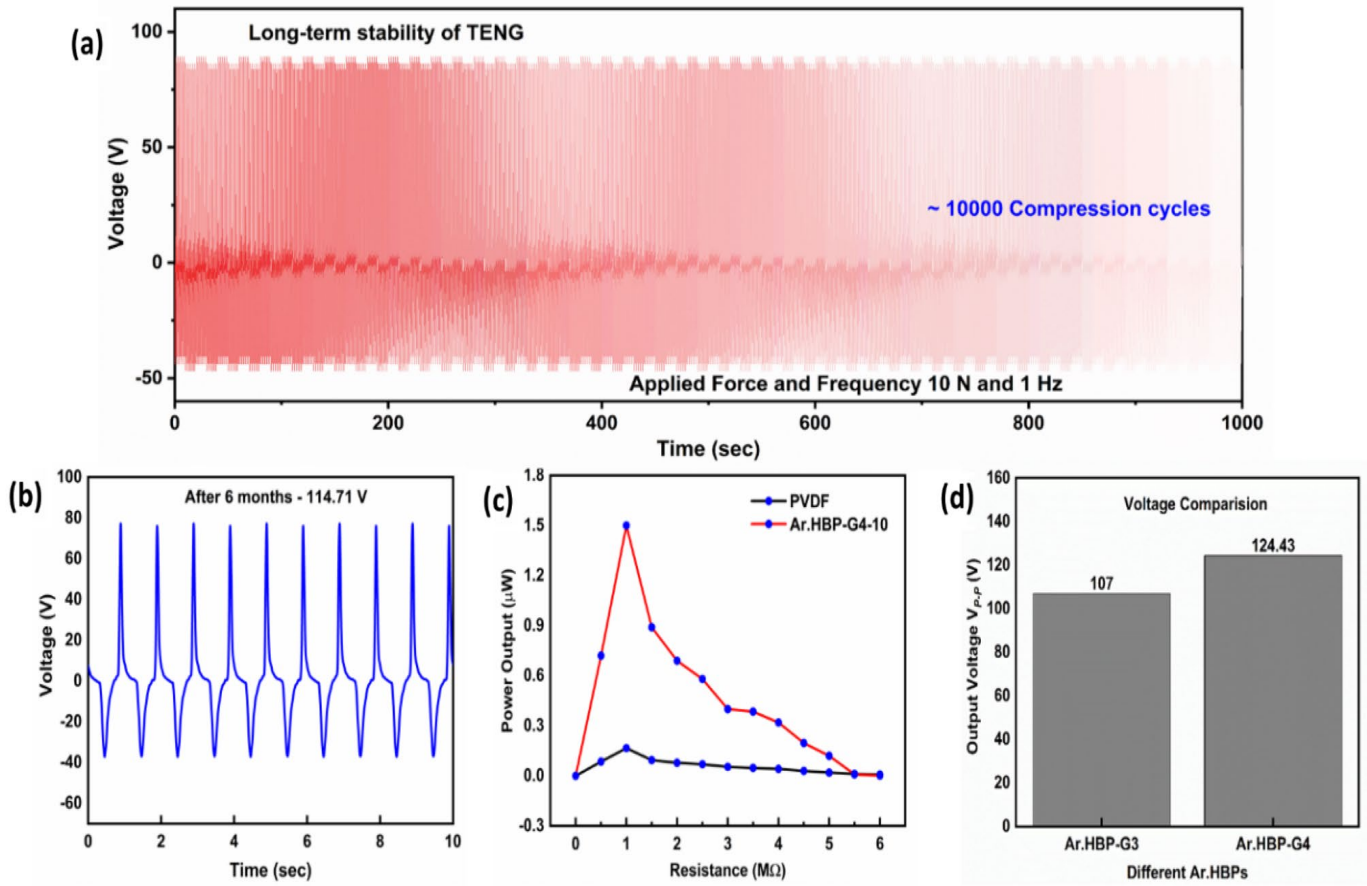
(**a**) Long-term stability of P-Ar.HBP-G4-10/TPU based TENG device for 10,000 cycles. (**b**) Durability of TENG device operated after 6 months. (**c**) Power output *vs* various load resistance and (**d**) Comparative output voltage plot of P-Ar.HBP-G3-10/TPU and P-Ar.HBP-G4-10/TPU based TENGs.

As shown in Fig. 5a, the output voltage of TENG was measured with good stability and the device operated continuously for more than 1000 s (∼ 10,000 cycles). No obvious decay was observed in the TENG performance during the ~ 10,000 compressing and releasing cycles, which indicates excellent mechanical stability without any significant decrease in the output voltage over long-term cycles. Further, the TENG response was tested after six months and found to be stable (Fig. 5b), which is beneficial for practical and commercial applications. The ability of the TENG to maintain high voltage output over a period of time is attributed to the improved nucleation effect of Ar.HBP-G4-10 aided by its highly branched structure, as already confirmed using FTIR and XRD data.

In order to measure the optimal load resistance and the output power from the TENG, various external load resistances were connected in parallel with the nanogenerator, and the output voltages were measured. Load resistances on electrical outputs ranging from 50 kΩ to 6 MΩ have been investigated. The output power was calculated using the following equation

$${\text{P}}=\frac{{V}^{2}}{R}$$

Figure 5c indicates that the power increased and subsequently reduced as the load resistance increased. With an external load resistance of 1 MΩ, the P-Ar.HBP-G4-10/TPU based TENG device depicts a maximum power equal to 1.51 μW corresponding to an output voltage of 124.4 V whereas in the case of PVDF/PU based TENG, it shows maximum power equal to 0.16 μW corresponding to an output voltage of 12.9 V. Figure 5d indicates the voltage comparison between P-Ar.HBP-G3-10/TPU and P-Ar.HBP-G4-10/TPU based TENGs. The 16% increase in higher output voltage generated using Ar.HBP-G4 compared to Ar.HBP-G3 is attributed to the increased branching structure and the availability of higher number of terminal OH groups in Ar.HBP-G4 that could have influenced the electron affinity of the TN layer, which in turn resulted in higher voltage output from the TENG device.

### Wearable and medical applications

**Figure 6:**
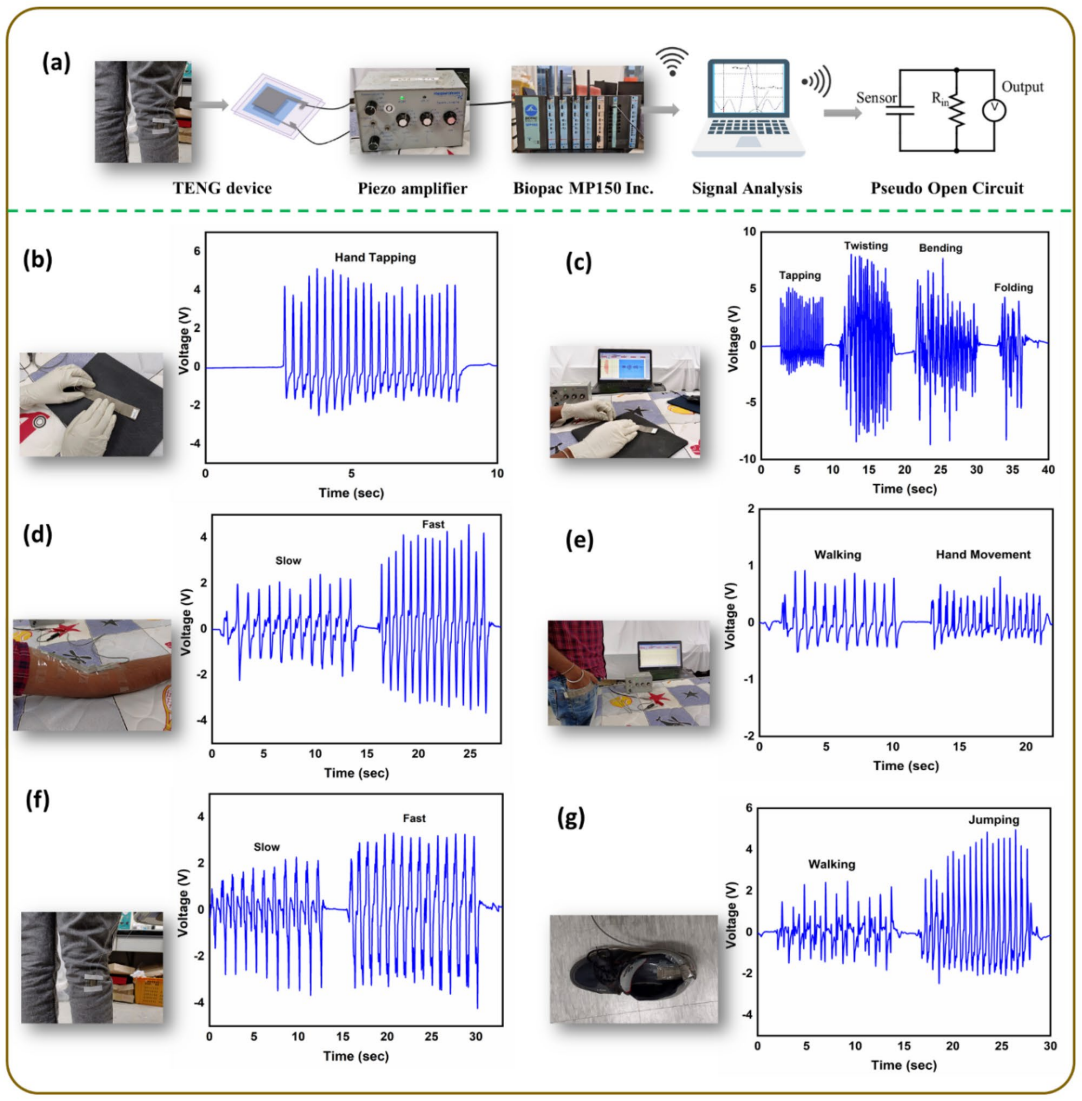
Real-time human health monitoring using P-Ar.HBP-G4-10/TPU based TENG: (**a**) Measurement components and pseudo open circuit diagram. Sensitivity efficiency of the TENG under (**b**) Normal hand tapping, (**c**) Various physical deformations (tapping, twisting, bending and folding), (**d**) Joint bending when device is placed at upper arm, and (**e**–**g**) Human motion detection and differentiation (hand movement, leg movement, walking and jumping) when the device is placed on cloth pocket, leg and shoe insole, respectively.

In the ambient environment, mechanical energy is abundant and the majority is spent in our daily activities, which can be harvested using TENGs. Figure 6a shows schematic representation of the general circuit used for human health monitoring measurements with Biopac MP150. Before evaluating a real-time wearable application, the sensitivity of P-Ar.HBP-G4-10/TPU TENG is determined by subjecting it to different body movements (Fig. 6b–g). When the TENG device is hand-tapped, it generated 7.65 V which confirms its sensitivity to normal human motion (Fig. 6b). The TENG device is also capable of precisely detecting finger tapping, twisting, bending and folding movements. The TENG generated 9 V at most when it is tapped, 15 V when twisted, 13 V when bent and 9 V when folded (Fig. 6c). When the device kept on the upper arm, it shows 4 V during slow motion and 7.6 V during fast motion (Fig. 6d). When the device is placed on the side pocket (Fig. 6e), it generated 1.31 V when the person is walking and 1.35 V when the hand was inserted and removed from the pocket as seen clearly from Video S1 of supplementary material. Similarly, when the device is fixed on the leg (Fig. 6f), the TENG generated 6 V and 6.9 V for slow and fast leg movement, respectively. When the device is fixed on the shoe sole (Fig. 6g), it generated maximum output voltage of 4.1 and 7 V for walking and jumping, respectively.

From the above results, it is favorable to use the P-Ar.HBP-G4-10/TPU based TENG in medical applications as a smart sensor, particularly for monitoring patients with coma and sleep apnea. To the best of our knowledge, there are no reports available on P-Ar.HBP-G4-10/TPU for bio-medical applications though reports are available on PVDF based TENGs^3,10^. Based on the data reported in the present study, P-Ar.HBP-G4-10/TPU is more effective in identifying and distinguishing various physical activities of humans, and has potential for applications in monitoring human actions and as a sustainable power source.

## Conclusions

Electrospun P-Ar.HBP-G4 NFs obtained by blending PVDF with Ar.HBP-G4 (0–40 wt-%) were studied for their enhancement in *β*-crystalline phase content using FT-IR and XRD measurements. From FE-SEM images, P-Ar.HBP-G4 electrospun NFs exhibits an evenly distributed fibrous morphology. A TENG device was successfully fabricated using P-Ar.HBP-G4 and melt-blown TPU as TN and TP layer, respectively. Compared to the 12.9 V output voltage (*V*_OC_) generated using pristine PVDF/TPU based TENG, the P-Ar.HBP-G4-10/TPU based TENG generated significantly higher output voltage of 124.4 V, which is an increase of 9.68 times. Also, under the constant frequency (1 Hz) and load (9.8 N), the TENG can efficiently operate 45 LEDs and also three different colored LEDs. The 22 *μ*F capacitor initially took 750 s to charge 3.7 V and is powered for 5 s. From the above results, the P-Ar.HBP-G4-10/TPU based TENG device has amply demonstrated real-time identifying and distinguishing various physical activities of humans, especially for monitoring patients with coma and sleep apnea. In conclusion, the fabricated TENG can be efficiently used in energy harvesting, wearable and biomedical applications.

### Supplementary Information

Supplementary Legends.

Supplementary Video 1.

Supplementary Figures.

## Data Availability

The data that support the findings of this study are available from the corresponding author upon reasonable request.

## References

[1] So MY, Xu B, Li Z, Lai CL, Jiang C (2023). Flexible corrugated triboelectric nanogenerators for efficient biomechanical energy harvesting and human motion monitoring. Nano Energy.

[2] Fahad MM (2022). Polysomnographic observation using triboelectric pressure sensor composed of polymer-pairs having coarse surface. Fibers Polym..

[3] Prasad G, Graham SA, Yu JS, Kim H, Lee DW (2023). Investigated a PLL surface-modified Nylon 11 electrospun as a highly tribo-positive frictional layer to enhance output performance of triboelectric nanogenerators and self-powered wearable sensors. Nano Energy.

[4] Gunasekhar R, Anand Prabu A (2023). Polyvinylidene fluoride/aromatic hyperbranched polyester 2nd generation based triboelectric sensor for polysomnographic and health monitoring applications. Sensors Actuators A Phys..

[5] Gajula P (2023). Fabrication of a silicon elastomer-based self-powered flexible triboelectric sensor for wearable energy harvesting and biomedical applications. ACS Appl. Electron. Mater..

[6] Liu Z, Zhao Z, Zeng X, Fu X, Hu Y (2019). Nano energy expandable microsphere-based triboelectric nanogenerators as ultrasensitive pressure sensors for respiratory and pulse monitoring. Nano Energy.

[7] Shrestha K (2023). Intermediate nanofibrous charge trapping layer-based wearable triboelectric self-powered sensor for human activity recognition and user identification. Nano Energy.

[8] Lai YC (2017). Single-thread-based wearable and highly stretchable triboelectric nanogenerators and their applications in cloth-based self-powered human-interactive and biomedical sensing. Adv. Funct. Mater..

[9] Zhang Q (2022). Wearable triboelectric sensors enabled gait analysis and waist motion capture for IoT-based smart healthcare applications. Adv. Sci..

[10] Graham SA (2022). Biocompatible electrospun fibers-based triboelectric nanogenerators for energy harvesting and healthcare monitoring. Nano Energy.

[11] Larcher D, Tarascon JM (2015). Towards greener and more sustainable batteries for electrical energy storage. Nat. Chem..

[12] Avanish Babu T, Madhuri W (2022). A hybrid microwave sintered PZT composite as a flexible piezoelectric nanogenerator. RSC Adv..

[13] Shi K, Sun B, Huang X, Jiang P (2018). Synergistic effect of graphene nanosheet and BaTiO_3_ nanoparticles on performance enhancement of electrospun PVDF nanofiber mat for flexible piezoelectric nanogenerators. Nano Energy.

[14] Sathiyanathan P (2021). Piezoelectric-piezocapacitive hybrid sensor based on electrospun poly(vinylidene fluoride)-poly(octafluoropentyl acrylate)-sulphonated poly(phenylene sulfide) blend nanofiber. Sensors Actuators A Phys..

[15] Shehata N (2022). Stretchable nanofibers of polyvinylidenefluoride (PVDF)/thermoplastic polyurethane (TPU) nanocomposite to support piezoelectric response via mechanical elasticity. Sci. Rep..

[16] Parangusan H, Ponnamma D, Al-Maadeed MAA (2018). Stretchable electrospun PVDF-HFP/Co-ZnO nanofibers as piezoelectric nanogenerators. Sci. Rep..

[17] Gunasekhar R, Prabu AA (2023). Fabrication of 1st generation aromatic hyperbranched polyester/ polyvinylidene fluoride blended electrospun composites to enrich the piezoelectric performance. J. Polym. Res..

[18] Wang X, Yang Y (2017). Effective energy storage from a hybridized electromagnetic-triboelectric nanogenerator. Nano Energy.

[19] Shao H (2017). Multifunctional power unit by hybridizing contact-separate triboelectric nanogenerator, electromagnetic generator and solar cell for harvesting blue energy. Nano Energy.

[20] Zi Y (2015). Triboelectric-pyroelectric-piezoelectric hybrid cell for high-efficiency energy-harvesting and self-powered sensing. Adv. Mater..

[21] Moalla R (2017). Huge gain in pyroelectric energy conversion through epitaxy for integrated self-powered nanodevices. Nano Energy.

[22] Zhang N (2016). A wearable all-solid photovoltaic textile. Adv. Mater..

[23] Lee MR (2009). Solar power wires based on organic photovoltaic materials. Science.

[24] Lin Z (2017). Triboelectric nanogenerator enabled body sensor network for self-powered human heart-rate monitoring. ACS Nano.

[25] Yi F (2015). Stretchable-rubber-based triboelectric nanogenerator and its application as self-powered body motion sensors. Adv. Funct. Mater..

[26] Wang Y, Yang Y, Wang ZL (2017). Triboelectric nanogenerators as flexible power sources. NPJ Flex. Electron..

[27] Zhu Y (2016). A flexible and biocompatible triboelectric nanogenerator with tunable internal resistance for powering wearable devices. Sci. Rep..

[28] Wang X (2016). A flexible triboelectric-piezoelectric hybrid nanogenerator based on P(VDF-TrFE) nanofibers and PDMS/MWCNT for wearable devices. Sci. Rep..

[29] Kurakula A, Graham SA, Paranjape MV, Yu JS (2023). Triboelectric film with electrochemical surface modification for multiple mechanical energy harvesting with high storage efficiency and sensing applications. ACS Appl. Electron. Mater..

[30] Manchi, P., Graham, S. A., Patnam, H., Paranjape, M. V. & Yu, J. S. rGO-ZnSnO_3_ Nanostructure-embedded triboelectric polymer-based hybridized nanogenerators. *Adv. Mater. Technol.* **7**, 2101460 (2022).

[31] Dudem B (2022). Wearable triboelectric nanogenerator from waste materials for autonomous information transmission via morse code. ACS Appl. Mater. Interfaces.

[32] Lin MF, Chang KW, Lee CH, Wu XX, Huang YC (2022). Electrospun P3HT/PVDF-HFP semiconductive nanofibers for triboelectric nanogenerators. Sci. Rep..

[33] Lett, R., Kim, M. P., Um, D. S., Shin, Y. E. & Ko, H. High‑performance triboelectric devices via dielectric polarization: A review. *Nanoscale Res. Lett.* **16**, 35 (2021). 10.1186/s11671-021-03492-4 PMC7881083 33580327

[34] Zou H (2020). Quantifying and understanding the triboelectric series of inorganic non-metallic materials. Nat. Commun..

[35] Kim, D. W., Lee, J. H., Kim, J. K. & Jeong, U. Material aspects of triboelectric energy generation and sensors. *NPG Asia Mater.* **12**, 6 (2020).

[36] Paquin F, Rivnay J, Salleo A, Stingelin N, Silva C (2015). Multi-phase semicrystalline microstructures drive exciton dissociation in neat plastic semiconductors. J. Mater. Chem. C.

[37] Wang S, Xie Y, Niu S, Lin L, Wang ZL (2014). Freestanding triboelectric-layer-based nanogenerators for harvesting energy from a moving object or human motion in contact and non-contact modes. Adv. Mater..

[38] Maity K, Garain S, Henkel K, Schmeißer D, Mandal D (2020). Self-powered human-health monitoring through aligned PVDF nanofibers interfaced skin-interactive piezoelectric sensor. ACS Appl. Polym. Mater..

[39] Sharafkhani S, Kokabi M (2021). High performance flexible actuator: PVDF nanofibers incorporated with axially aligned carbon nanotubes. Compos. Part B Eng..

[40] Kim DB (2022). Weave-pattern-dependent fabric piezoelectric pressure sensors based on polyvinylidene fluoride nanofibers electrospun with 50 nozzles. NPJ Flex. Electron..

[41] Van Ngoc H, Kang DJ (2016). Flexible, transparent and exceptionally high power output nanogenerators based on ultrathin ZnO nanoflakes. Nanoscale.

[42] Lin ZH (2013). Enhanced triboelectric nanogenerators and triboelectric nanosensor using chemically modified TiO_2_ nanomaterials. ACS Nano.

[43] Wang J (2015). A flexible fiber-based supercapacitor–triboelectric-nanogenerator power system for wearable electronics. Adv. Mater..

[44] Chen A, Zhang C, Zhu G, Wang ZL (2020). Polymer materials for high-performance triboelectric nanogenerators. Adv. Sci..

[45] Huang T (2015). Human walking-driven wearable all-fiber triboelectric nanogenerator containing electrospun polyvinylidene fluoride piezoelectric nanofibers. Nano Energy.

[46] Li Y (2022). Advances in electrospun nanofibers for triboelectric nanogenerators. Nano Energy.

[47] Wang Y, Yokota T, Someya T (2021). Electrospun nanofiber-based soft electronics. NPG Asia Mater..

[48] Gunasekhar R (2023). Polyvinylidene fluoride/aromatic hyperbranched polyester of third-generation-based electrospun nanofiber as a self-powered triboelectric nanogenerator for wearable energy harvesting and health monitoring applications. Polymers (Basel)..

[49] Prasad G, Sathiyanathan P, Prabu AA, Kim KJ (2017). Piezoelectric characteristics of electrospun PVDF as a function of phase-separation temperature and metal salt content. Macromol. Res..

[50] Sathiyanathan P, Dhevi DM, Prabu AA, Kim KJ (2019). Electrospun polyvinylidene fluoride-polyoctafluoropentyl acrylate-hydroxyapatite blend based piezoelectric pressure sensors. Macromol. Res..

