# Publisher Correction: Electrospun PVDF/aromatic HBP of 4th gen based flexible and self‑powered TENG for wearable energy harvesting and health monitoring

**DOI:** 10.1038/s41598-024-52371-2

**Published:** 2024-01-19

**Authors:** Ramadasu Gunasekhar, Mohammad Shamim Reza, Kap Jin Kim, Arun Anand Prabu, Hongdoo Kim

**Affiliations:** 1 Department of Chemistry, School of Advanced Sciences, Vellore Institute of Technology, Vellore, 632014 India; 2 Department of Advanced Materials Engineering for Information and Electronics, College of Engineering, Kyung Hee University, Yongin-Si, Gyeonggi-do 17104 Republic of Korea

Correction to: *Scientific Reports* 10.1038/s41598-023-50231-z, published online 19 December 2023

In the original version of this Article, Arun Anand Prabu was incorrectly affiliated with ‘Department of Advanced Materials Engineering for Information and Electronics, College of Engineering, Kyung Hee University, Yongin-si, Gyeonggi-do 17104, Republic of Korea.’

The correct Affiliation is listed below:

‘Department of Chemistry, School of Advanced Sciences, Vellore Institute of Technology, Vellore 632014, India.’

The original Article has been corrected.

