# Supplementary material for: Electrospun PVDF/aromatic HBP of 4th gen based flexible and self-powered TENG for wearable energy harvesting and health monitoring
Source: Sci Rep. 2023 Dec 19;13:22645. doi: 10.1038/s41598-023-50231-z (PMC10730851; doi:10.1038/s41598-023-50231-z)
Supplement: Supplementary file 1 — Supplementary Legends. [file 41598_2023_50231_MOESM1_ESM.docx]

# Supplementary information

The following information is provided in supplementary material file.

- Figure S1. Schematic for synthesis of Ar.HBP-G4,
- Figure S2. Characterization of Ar.HBP-G4 (a). FTIR spectra and (b). 1H-NMR spectra,
- Figure S3. Triboelectric series chart of different materials and
- Video S1. Demonstration of the TENG performance for pant pocket sensor.
