# Supplementary material for: Electrospun PVDF/aromatic HBP of 4th gen based flexible and self-powered TENG for wearable energy harvesting and health monitoring
Source: Sci Rep. 2023 Dec 19;13:22645. doi: 10.1038/s41598-023-50231-z (PMC10730851; doi:10.1038/s41598-023-50231-z)
Supplement: Supplementary file 3 — Supplementary Figures. [file 41598_2023_50231_MOESM3_ESM.docx]

**Appendix A. Supplementary Material**

**Electrospun PVDF/Aromatic HBP of 4^th^ Gen based Flexible and Self-powered TENG for Wearable Energy Harvesting and Health Monitoring**

Ramadasu Gunasekhar ^a,†^, Mohammad Shamim Reza ^b,†^, Kap Jin Kim ^b^, Arun Anand Prabu ^a,^* and Hongdoo Kim ^b,^*

^a^ Department of Chemistry, School of Advanced Sciences, Vellore Institute of Technology, Vellore 632014, India; gunasekhar.r2019@vitstudent.ac.in (R.G.)

^b^ Department of Advanced Materials Engineering for Information & Electronics, College of Engineering, Kyung Hee University, Yongin-si 17104, Gyeonggi-do, Republic of Korea; reza13@khu.ac.kr (M.S.R.); kjkim@khu.ac.kr (K.J.K.)

***** Correspondence: [anandprabu@vit.ac.in](mailto:anandprabu@vit.ac.in) (A.A.P.); [hdkim@khu.ac.kr](mailto:hdkim@khu.ac.kr) (H.K.)

^,†^ R.G. and M.S.R. have contributed equally to this work.

**Figure S1:** Schematic for synthesis of Ar.HBP-G4.





**
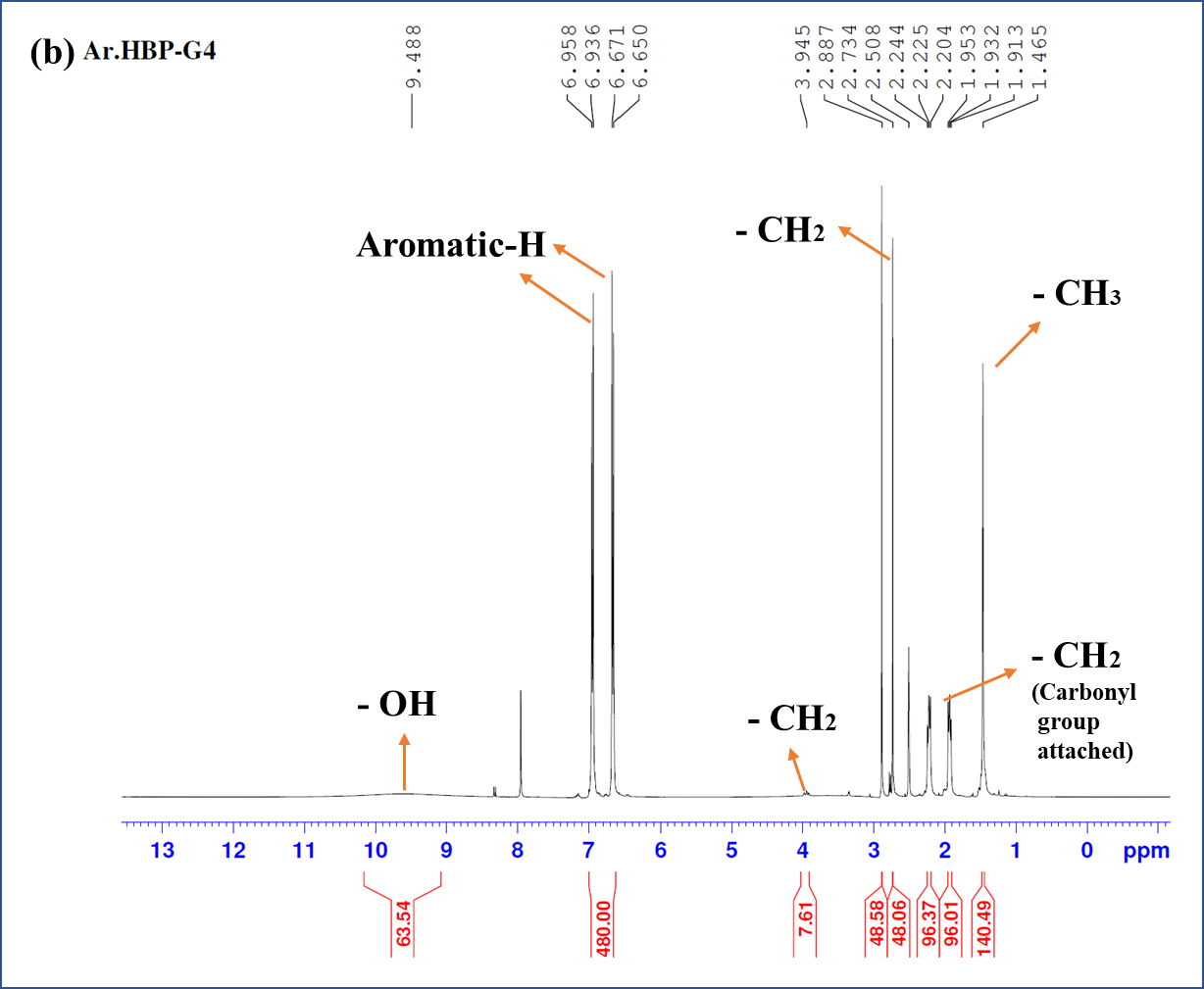
**

**Figure S2:** Characterization of Ar.HBP-G4 (a). FTIR spectra and (b). ^1^H-NMR spectra.

**Triboelectric series chart:**


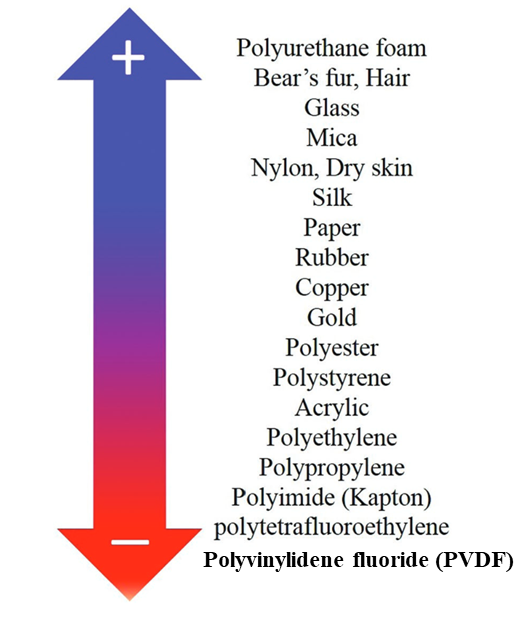


**Figure S3:** Triboelectric series chart of different materials.
